# Contact Allergy Induced by Mango (*Mangifera indica*): A Relevant Topic?

**DOI:** 10.3390/medicina57111240

**Published:** 2021-11-13

**Authors:** Elena Camelia Berghea, Mihai Craiu, Selda Ali, Sabina Loredana Corcea, Roxana Silvia Bumbacea

**Affiliations:** 1 Pediatrics Department, “Carol Davila” University of Medicine and Pharmacy, 050474 Bucharest, Romania or bcamelia@gmail.com (E.C.B.); mcraiu@yahoo.com (M.C.); 2 Department of Pediatrics, “Marie Curie” Emergency Children’s Hospital, 077120 Bucharest, Romania; 3 Department of Pediatrics, “Alfred Rusescu” Clinical Pediatric Hospital, Institute of Mother and Child Health, 020395 Bucharest, Romania; 4 Allergology Department, “Carol Davila” University of Medicine and Pharmacy, 050474 Bucharest, Romania; Roxana.bumbacea@umfcd.ro; 5 Department of Allergology and Clinical Immunology, “Carol Davila” Nephrology Clinical Hospital, 010731 Bucharest, Romania; 6 Pharmacology and Pharmacotherapy Department, “Carol Davila” University of Medicine and Pharmacy, 050474 Bucharest, Romania; sabinacorcea@yahoo.com; 7 Novomedica Center of Excellence, 030167 Bucharest, Romania

**Keywords:** mango hypersensitivity, contact allergy, contact dermatitis, patch testing, urushiol

## Abstract

Introduction: The most common clinical manifestation of mango allergy is contact dermatitis, which can be localized or systemic. The sensitising substances that have long been suspected are alk(en)yl catechols and/or alk(en)yl resorcinols. Methods: We reviewed the original articles published on Pubmed, Embase and Cochrane Library before 15 September 2021, on the topic of contact allergy induced by mango and we synthesized the key data. Results: We found 12 case reports and four case series, with a total of 37 patients. Only seven of these cases were reported in patients from mango-cultivating countries, the other 30 were from countries where mango cultivation does not occur, and 26 were also from countries where poison ivy/oak are commonly found. We found that contact dermatitis may occur on the first exposure to mango due to previous sensitisation to urushiol-containing plants. The diagnosis was confirmed by patch testing in some of the cases. There was great heterogeneity between the reagents used. Conclusion: Mango fruit is frequently consumed, but mango induced contact dermatitis, the main hypersensitivity reaction induced by mango, is rare. Further data is necessary for a better understanding of sensitising substances and, consecutively, standardization of patch test reagents.

## 1. Introduction

Mangoes are consumed almost everywhere in the world; nevertheless, few reports of hypersensitivity reactions (both immediate and delayed) have been mentioned in the literature. From the first allergic reaction to mango reported in 1939 [1] to date, only a small number of cases has been published.

Mango allergy may occur as type I hypersensitivity reactions ranging from erythema, hives, angioedema, wheezing or oral allergy syndrome to severe anaphylactic reactions, or as type IV hypersensitivity reaction such as contact dermatitis. The latter appears to be more prevalent.

The present manuscript is a compilation of essential concepts on physiopathology, clinical manifestations, and allergenic profile of mango (*Mangifera indica*) and a concise review of mango-induced contact dermatitis published cases.

## 2. *Mangifera indica*-General Data

*Mangifera indica,* commonly known as mango, is a flowering plant species native to the Indo-Burmese region and has been cultivated for around 4000 years; it is a member of the Anacardiaceae family, from the Sapindales order [2]. Other key members of the Anacardiaceae family are poison ivy, poison oak, sumac, pistachio and cashew. Mango fruit is referred to as the “king of fruits” or “fruit of the gods” and is the fifth most widely consumed fruit in the world [2].

*M. indica* is an evergreen tree cultivated in tropical and subtropical climate regions such as Asia, some countries in Europe (Spain) and South America (Mexico, Brazil) and as far as Australia. Half of the mangoes produced worldwide originate from India. They are the widest spread fruit crop within the subcontinent of India, which ranks first among the world’s mango producing countries [3].

The mango fruit is a drupe, consisting of the peel (epicarp) that covers the pulp (mesocarp which encases a fibrous, hard stone (endocarp) inside of which is found a single seed [4,5].

Mango is consumed throughout the year, both in fresh forms (plain fruit, juices, smoothies) and in processed forms (pickled mango, compotes, jams, purees, dehydrated or frozen). It is also used in indigenous and traditional medicines for its anti-oxidant, anti-inflammatory, anti-viral, anti-bacterial, anti-atherosclerotic, anti-diabetic and anti-cancer properties [6].

## 3. Pathophysiology and Clinical Manifestations of Mango Hypersensitivity

Two types of hypersensitivity responses to mango have been described with distinct clinical manifestations that include immediate hypersensitivity and delayed hypersensitivity reactions.

Immediate hypersensitivity reaction to mango is an IgE-mediated reaction (type I hypersensitivity as classified by Gell & Coombs), which involves mast cell degranulation in individuals sensitised to its antigens. The release of inflammatory mediators, such as proteases, lysosomal enzymes and particularly histamine, leads to vasodilation, eosinophils and leukocytes recruitment, resulting in inflammation and possibly bronchoconstriction [7].

Symptoms may be limited to the contact area or may be systemic including erythema, urticaria, angioedema, wheezing, coughing, sneezing, nasal congestion, and bronchospasm, some leading to severe anaphylaxis. Even though rare, cases of anaphylactic shock related to mango consumption have been reported in the literature [8,9].

A review of the immediate hypersensitivity reactions induced by mango [10] showed that atopic patients are more likely to develop type I hypersensitivity to mango (8 out of the 10 cases documented being atopic) [11].

Oral allergy syndrome (OAS) to mango is defined as an IgE antibody-mediated allergic reaction that occurs in response to eating fresh mango that typically develops in patients with clinical sensitisation to pollen. OAS is not a distinct food allergy; symptoms rather arise due to cross-reactivity between pollen and mango. The main sensitisation is to pollen [12]. The common manifestations of OAS develop just a few minutes after the fruit intake and include itching or burning sensation of the mouth, including the lips, the tongue and the throat, which may be accompanied by swelling. Vomiting, diarrhea, and occasionally anaphylaxis may occur. However, a few cases with late onset of symptoms but similar clinical patterns have been described in the literature [1]. Mango may cross-react with other Anarcardiaceae family members, particularly poison ivy, poison oak and cashew nut, and with various respiratory allergens (including mugwort pollen and birch pollen), foods and latex [13,14].

The delayed hypersensitivity reaction to mango (type IV hypersensitivity reaction in Gell & Coombs classification) is mediated by T helper 1 cells. The pathogenic response involves a local inflammatory response after the close contact with the antigen of mango fruit or with the bark of the mango tree. After the initial contact, the antigen is presented to T helper cells by macrophages, thereby T helper cells become sensitised and start to release cytokines and chemokines that enhance the inflammatory response, damaging the keratinocytes and causing epithelial tissue disruptions [7,15].

The most common clinical manifestation is contact dermatitis, which can be localised or systemic (disseminated) and is represented by rash, pruritus, eczema and blisters. Dermatitis may appear on the extremities after contact with mango fruit, peel, stem, sap, or even the tree itself [16,17]. Other cutaneous signs include lips and perioral eczematous lesions or periorbital edema. Usually, the onset of symptoms is within 8–12 h after the contact; they consist of rash and induration, followed by blister formation within 72 h.

Some cases presenting with allergic contact dermatitis to mango without previous exposure to the fruit or tree have been described [18]. Notably, the sensitisation may also occur through close contact with other family members such as poison ivy or poison oak [19].

## 4. Allergenic Profile and Significant Cross-Reactivity

Mango allergens have not been fully characterized because the allergenic composition of the fruit is not completely known. There are 334 different proteins that were found in mango peel, and 2855 in the fruit flesh and some of these proteins may have immunizing properties [20].

Only three mango allergens (two major allergens and one minor allergen, a profilin) have been identified so far using sera from patients with mango IgE-mediated sensitisation. These are mentioned in Table 1 based on their designation, made using the first three letters of the genus followed by the first letter of the species and the identified protein group’s number [21].

Despite enzymatic degradation, mechanical fragmentation, and heating, during peeling or pasteurization, mango allergens were found to be relatively stable throughout technological processing. Mango purées and nectar extracts showed no significant decrease in allergenicity [13,22]. The heat and processing resistance of mango profilin appears to be remarkable [22].

Profilins, well-known pan-allergens, are proteins with highly conserved structure that can be found in all eukaryotic cells, are involved in processes related to cell motility and share sequence identities of more than 75%, even between members of distantly related organisms [23]. Mango profilin has demonstrated a high degree of cross-reactivity with birch pollen. Likewise, an isoform of mango profilin (Man i 3.02) appears very similar to a profilin found in pear, peach and apple [24].

Hevein (Hev b 6.02; a class I chitinase-like protein) is the allergen that causes most latex–fruit syndrome cases; its homologous proteins were found in mango along with avocado, chestnut, banana, kiwi, tomato, passion fruit and papaya [25].

Although significant cross-reactivity between certain species from the family and genus (for example, pistachio and cashew nut) is possible, it is not commonly observed [26]. All the above mentioned allergens are involved in immediate hypersensitivity reactions.

The sensitising substances that have long been suspected of inducing delayed hypersensitivity reactions to mango are alk(en)yl catechols and/or alk(en)yl resorcinols [27,28,29].

The term “Mangol” refers to the three resorcinol derivatives: hepta-decadienylresorcinol (I), hepta-decenylresorcinol (II), and penta-decylresorcinol (III), that have been identified as mango “allergens” [30]. These are distributed within the skin, bark, leaves and in the first 5 mm of mango’s pulp and have been shown to elicit positive patch test reactions in mango sensitised patients. The mango plant’s leaves, stems, and pericarp also contain other compounds that were thought to be sensitisers, including cardol, beta-pinene, and limonene [30].

The “Mangol” has the potential to cross-react with urushiol, therefore mango contact dermatitis has been associated with a history of poison ivy and poison oak exposure, which can induce sensitisation via urushiol. As a result, mango allergic contact dermatitis may occur in people who are exposed to mango for the first time through direct contact with mango tree and fruit or after ingestion of mango fruit, especially unpeeled mango [28,31]. This cross-reactivity was highlighted by many clinical studies and case reports, but it has not been sustained by others [32]. To sum up, some patients may or may not show allergic contact dermatitis cross-reactivity between urushiol and mango alkyl-resorcinols.

## 5. Mango Allergic Contact Dermatitis Diagnosis

Diagnosis of mango sensitivity is guided by the clinical presentation. In order to confirm the suspicion of mango contact dermatitis, patch tests can be done with various mango extracts, prepared from mango peel, pulp, leaf, sap, stem, and with resorcinol fractions or urushiol. Usually contact reactions to mango are induced by direct contact with the peel of the fruit, but rare cases of mango pulp contact dermatitis have been reported, thus both peel and pulp should be tested in this context [31]. In cases where history is not clear, one may add prick-to-prick testing in order to rule out type I hypersensitivity reaction.

There is heterogeneity regarding testing reagents; patch testing can be adapted depending on clinical presentation and history of the patient, as is shown in the reviewed published cases.

## 6. Data Sources and Results

We reviewed the relevant published literature on Pubmed, Embase, Cochrane Library on the topic of contact allergy induced by mango published before 15 September 2021 and synthesized the key data. Medical Subject Heading (MeSH) and key-words were used together, including mango, contact hypersensitivity, contact allergy. This approach was also combined with a manual search of references in all selected studies.

We found 12 case reports and four case series, with a total of 37 patients diagnosed with mango contact dermatitis. The most important features of the 37 selected cases are summarized in Table 2.

We did not include in our analysis a study assessing different patch reagents (mango peel or pulp in aqueous or ether solution) in patients with “itchy dermatitis on the face and hands possibly due to eating mango” and a control group consisting of patients with non-eczematous diseases of the skin. The article did not offer any other information about the patients who tested positive on patch tests [33].

## 7. Discussion

Out of the 37 cases described in Table 2, only seven cases were reported from mango-cultivating countries: Australia, Spain and Thailand. The remainder were documented from countries where mango cultivation does not occur (25 from the USA, three from Japan, and two cases, each from Canada and France). Furthermore, 26 out of these 30 cases were also from countries where poison ivy and poison oak are commonly found (USA and Canada), five of which had previous known sensitisation to these plants. Considering the cross reactivity between these plants and mango, all belonging to the same family of Anacardiaceae, a previous sensitisation may explain the increased number of contact dermatitis cases due to mango in USA and Canada.

Contact dermatitis can occur on the first exposure to mango due to previous sensitisation to urushiol-containing plants, primarily those in the Anacardiaceae family. Seven patients had previous known sensitisation to poison ivy, poison oak and Lithracea caustica [16,17,31,36,38,40,41]. There was one case where the patient had no known sensitisation to urushiol containing plants, but nevertheless tested positive for urushiol 0.01% in petrolatum at patch testing [28]. A report of 17 individuals from the United States, who developed a rash after participating in mango picking at a summer camp in Israel, supports the observation that mango contact dermatitis can occur at first mango exposure. In contrast, their Israeli fellows, who had never been exposed to poison ivy/oak and from a mango-cultivating region, did not present any skin lesion while engaged in the same activity. The authors hypothesized that the presence of common compounds such as urushiol in poison ivy/oak sensitised the American individuals to mango [18].

Exposure to contact allergens found in mango can occur in different ways. Of the 30 patients described above, 23 had been in direct contact with the mango fruit, skin, stem, sap, or even the tree itself [16,17,18,28,36]. The remaining patients had ingested mango either peeled or unpeeled, fresh or processed in the form of mango gelato [1,28,29,31,34,35,37,38,39,40,41,42]. Patients who had been in direct contact with mango developed skin lesions at contact site, presenting as pruritic and erythematous rash, with occasional vesicles and bullae on the extremities or on the lips, and in the perioral area.

In case of mango ingestion, patients developed lesions consistent either with localised contact dermatitis (perioral skin lesions associated with occasional lip and facial edema), or with disseminated dermatitis (patients displaying skin lesions in areas that were not in contact with the fruit) [35,38,42]. It was previously thought that perioral lesions can develop if the fruit is eaten whole, without removal of the skin, thus the fruit could be enjoyed if peeled by another person [43]. However, Weinstein et al. had shown that the 5 mm of flesh under the skin contains enough sensitising compounds to induce dermatitis in sensitised patients [34]. This information should be kept in mind when preparing mango products for patch testing.

Disseminated contact dermatitis was suspected in the three cases where the patients presented diffuse pruritic rash in one case [38], recurrent eyelid edema with a bilateral thigh rash in another case [42] and vasculitis with palpable purpura described in the third case [35].

The time to onset of symptoms amongst the 37 cases ranges from a few hours (4–5 h) to several days (up to 9 days). Therefore, the clinical suspicion of a delayed reaction had to be confirmed by patch testing. This was done in 16 out of 37 patients and was positive in all of them. Unfortunately, in the available data there were insufficient details regarding the patch testing protocol. The allergy testing method was heterogenous, with patch testing using either mango skin, sap, leaf, stem, flesh or juice, without providing details on mango reagents’ preparation [1,16,29,31,34,35,39,40,42], urushiol 0.02% in one case [35] and two components of Mangol (hepta-decadienal resorcinol and hepta-decenyl resorcinol) from the Philippine mango, adjusted in 0.05% concentration in petrolatum in two other cases [28]. Patch test with mango juice was negative in both cases when it was performed.

Even so, there is another report in which the authors tested different preparations of mango in aqueous solution and ether solution and concluded that a conventional preparation of 10% mango extract in ether might be used to test suspected individuals for mango contact dermatitis [33].

Skin prick tests were done only in four cases since an immediate hypersensitivity was not the main suspected diagnosis. Skin prick tests with mango were negative in three out of these four cases [31,35,39,40].

Nevertheless, there was one reported case in which both type I and type IV hypersensitivity were suspected [40]. The patient, who had a history of Anacardiaceae plants hypersensitivity, developed perioral eczematous lesions immediately after contact with mango flesh. Both prick-by-prick test (type I hypersensitivity) and delayed patch testing (type IV hypersensitivity) using mango flesh provided positive results.

## 8. Final Remarks

Mango-induced allergic contact dermatitis remains a rare clinical condition; however, we must consider it in patients with suggestive exposure history and clinical presentation. Contact with the mango fruit, skin, stem, sap, or even the tree itself may induce localised or systemic symptoms. When the history is suggestive, patch testing with different parts of the mango plant, urushiol and resorcinol may significantly contribute to a correct diagnosis. Until today there have been few published cases. We reviewed four studies and 12 case reports that totalized 37 patients. The reagents used for patch testing were heterogenous, using different parts of the fruit or of the tree. Although immediate hypersensitivity to mango is more prevalent in atopic patients, there are no data available on the presence of atopy in patients diagnosed with delayed hypersensitivity to mango. One must keep in mind that mango allergic contact dermatitis may occur on first contact with the fruit or other part of the plant, especially in people living in areas of the world where poison ivy or poison oak grow.

## Figures and Tables

**Table 1:** Mango allergens profile.

Allergen	Type	Molecular Weight	Protein Function
Man i 1	Major allergen	40 kDa	Unknown function
Man i 2	Major allergen	30 kDa	Unknown function
Man i 3	Minor allergen	Unknown	Profilin

**Table 2:** Published cases of mango-induced contact dermatitis.

Reference	Country	Patients Age/ Gender	Presenting Symptoms	Exposure	Time to Onset of Symptoms	Patch Test	Skin Prick Test to Mango	Other Sensitisations
Zakon, S. 1939 [1]	USA	29 yo Female	Erythematous and vesicular eruption on lips, chin and both cheeks and lip edema; nausea	Mango ingestion	24 h	Mango skin positive Mango juice negative	Unknown	Strawberries
		19 yo Female	Lip edema, perioral erythema with discrete vesicles and bullae	Mango ingestion	24 h	Mango skin positive Mango juice negative	Unknown	Unknown
Calvert, M. L. 1996 [16]	Australia	21 yo Female	Intensely pruritic linear papulo-vesicular lesions on lower legs, urticarial plaques on forearms	Picking mangoes	4 h	Mango sap, leaf, stem, skin-positive	Unknown	Poison ivy
		31 yo Female	Intensely pruritic confluent urticaria on the arms and abdomen	Mango tree contact	12 h	Mango sap, leaf, stem, skin-positive	Unknown	Unknown
		27 yo Female	Pruritic confluent urticaria on neck, acute eczematous plaques with bullae on arms	Picking mangoes	6 days	Mango sap, leaf, stem, skin-positive	Unknown	Unknown
		36 yo Male	Widespread acute eczematous and urticarial plaques	Mango tree contact	5 h	Mango sap, leaf, stem, skin-positive	Unknown	Unknown
Tucker, M. O. 1998 [17]	USA	27 yo Male	Pruritic and eczematous rash on his right leg	Resting his hand on the leg after peeling a mango	3 days	Unknown	Unknown	Poison oak and poison ivy
Oka, K. 2004 [28]	Japan	25 yo Female	Erythematous papular reaction around the lips, ear and right hand, periocular erythema and swelling	Peeling and eating mango	1 day	Hepta-decadienylresorcinol (0.05% PET) and hepta-decenylresorcinol (0.05% PET) positive	Unknown	Urushiol (0.01% PET)
		27 yo Female	Erythematous and vesiculous lesions on lower lip, chin and neck	Mango ingestion	9 days	Hepta-decadienylresorcinol (0.05% PET) positive	Unknown	Unknown
Weinstein, S. 2004 [34]	USA	22 yo Female	Pruritic erythema of the face, neck, and arms and periorbital edema	Peeled mangoes ingestion	Unknown	Mango skin and flesh-5 mm below the skin-positive	Unknown	Nickel, p-tertbutyl-phenol formaldehyde resin
Thoo, C. H. 2008 [35]	Australia	42 yo Female	Palpable purpuric eruption on abdomen, arms, legs and neck over a 4-week period	Fresh mango gelato ingestion	4 days	Mango skin and flesh-1cm below the skin-positive at 24 h	Mango skin and flesh negative	No
Wiwanitki, V. 2008 [29]	Thailand	42 yo Female	Patchy, pruritic erythema on the face and extremities and periorbital edema	Peeled mango ingestion	1 day	Mango skin and flesh positive	Unknown	Unknown
Trehan, I. 2010 [36]	USA	25 yo Male	Lip edema with pruritic rash; subcutaneous edema on the face and periorbital edema with ptosis	Peeling a mango with his teeth	2 days	Unknown	Unknown	Poison oak
Kim, A. S. 2015 [31]	USA	23 yo Male	Perioral erythematous and pruritic rash with lip edema; a pruritic and erythematous rash on the abdomen and medial thighs and bilateral periorbital edema	Mango ingestion and mango juice contact	1 day	Mango skin and flesh (2 cm below the skin)-positive	Mango skin and flesh negative	Poison oak
Miyazawa, H. 2018 [37]	Japan	27 yo Female	Lip oedema with crusts and erosions	Unpeeled mango ingestion	1 day	Urushiol 0.02%-positive	Unknown	Unknown
Yoo, M. 2019 [38]	USA	41 yo Male	Macular, pruritic rash on all extremities, inguinal area, chest and back	Mango ingestion	2 days	Unknown	Unknown	Poison ivy
O’Hern, K. 2020 [39]	USA	12 yo Male	Perioral, eczematous, well-demarcated crescent-shaped pruritic plaque and periocular erythematous papules (“mango slice” dermatitis)	Mango slices ingestion	Unknown	Mango skin and flesh-positive	Mango- negative	Pineapple, mixed fruits
Pesqué, D. 2021 [40]	Spain	51 yo Female	Pruritic, eczematous cheilitis, perioral dry eczematous patches (Protein contact dermatitis)	Mango ingestion	Unknown	Mango flesh-positive	Mango flesh positive	Lithracea caustica (Anacardiaceae family)
Alipour Tehrany Y. 2021 [41]	Canada	9 yo Male	Well-demarcated erythematous-squamous plaques on the nose, peri-oral region, chin and cheeks and eyelid edema	Unpeeled mango ingestion	1 day	Unknown	Unknown	Poison ivy
Raison-Peyron, N. 2021 [42]	France	30 yo Female	Vesicular cheilitis, right eyelid edema and erythematous rash on thighs	Mango ingestion	Unknown	Mango skin and flesh positive	Unknown	Tea tree oil, limonene hydroxy-peroxide
Hershko, K. 2005 [18]	USA	17 patients (11 males; 6 females)	Widespread eczematous urticarial rash	Mango picking	Unknown	Unknown	Unknown	Poison oak and poison ivy (possible)

yo = years old.

## Data Availability

Not applicable.
